# Socioeconomic characteristics and the burden of non-communicable diseases: a cross-sectional analysis of Tanzanian households, 2021

**DOI:** 10.11604/pamj.2025.52.127.47787

**Published:** 2025-11-26

**Authors:** Winfrida Kaaya, Alphoncina Kagaigai, Alma Damasy, Nathanael Sirili

**Affiliations:** 1 Department of Development Studies, Muhimbili University of Health and Allied Sciences, Dar es Salaam, Tanzania,; 2 Department of Emergency Medicine, Muhimbili University of Health and Allied Sciences, Dar es Salaam, Tanzania

**Keywords:** Chronic diseases, socioeconomic status, cardiovascular health, health disparities, non-communicable diseases, health insurance

## Abstract

**Introduction:**

Non-Communicable Diseases (NCDs) pose a serious global health challenge. The burden of NCDs affects individuals across various demographic categories. Socioeconomic status (SES) has long been recognized as a determinant of health outcomes. Previous research suggests a complex interplay between SES and NCDs, with disparities in prevalence and disease management observed across different socioeconomic strata. This study aims to further examine the association between NCDs and socioeconomic characteristics to inform evidence-based interventions that can effectively mitigate the burden of NCDs.

**Methods:**

this study was a secondary analysis of a cross-sectional dataset collected in 2021 by the Tanzanian National Institute for Medical Research (NIMR). The original study assessed willingness and ability to pay for medical insurance. The current study employed quantitative approaches. Logistic regression is used to assess the association between SES and NCDs, adjusting for potential confounders including age, gender, family size, health insurance status, and place of residence. A backward elimination approach was used to retain variables with p < 0.2 in the final model. Socioeconomic status was assessed using an asset index generated through principal component analysis.

**Results:**

a total of 3,566 households were analyzed, with a mean age of 38.04 (SD: 11.76), 53.7% of whom were male. The overall prevalence of NCDs was 12.9%. Households in lower SES quintiles had higher NCD prevalence than those in higher SES quintiles, although this association was not statistically significant after adjustment. However, age, larger family size, and lack of health insurance remained statistically significant predictors of NCD prevalence.

**Conclusion:**

while a clear association was observed between lower SES and NCDs in the univariate analysis, this relationship lost statistical significance after adjusting for demographic and household characteristics. However, age, family size, and lack of health insurance remained significant predictors of NCD prevalence.

## Introduction

With the epidemiological transition, marked by a shift from infectious diseases and high mortality rates to longer life expectancy and a predominance of chronic conditions, non-communicable diseases (NCDs) are increasingly becoming a global threat [1]. Unlike infectious diseases, most NCDs result from a combination of genetic, physiological, environmental, and behavioral factors [1,2]. They significantly impact global health by causing long-term disability, reducing productivity, and increasing healthcare costs [2-6]. Non-communicable diseases have become the leading cause of mortality worldwide, accounting for approximately 71% of all deaths annually [4]. These conditions - including cardiovascular diseases, diabetes, cancers, and chronic respiratory diseases - disproportionately affect low- and middle-income countries (LMICs), where over 85% of premature NCD-related deaths occur [4]. In sub-Saharan Africa, the burden of NCDs is rapidly increasing and now contributes to more than 37% of all deaths in the region, driven by epidemiological transition, urbanization, changing lifestyles, and inadequate health infrastructure [7,8]. This shift in disease burden poses unique challenges for countries that are still struggling with infectious diseases, limited health infrastructure, and under-resourced health systems [9]. Despite lower overall mortality rates from NCDs compared to high-income settings, the region experiences higher rates of premature death and lower access to preventive and treatment services [1]. In Tanzania, NCDs are increasingly recognized as a significant public health concern. Recent data indicate rising trends in the prevalence of hypertension, diabetes, and cardiovascular conditions, particularly in urban areas [10,11]. These conditions contribute significantly to outpatient visits, hospital admissions, and long-term economic costs to households and the health system.

According to the mortality patterns among hospital deaths in Tanzania in the 2006-2015 period, approximately 33% of hospital deaths are attributed to NCDs [12]. While the Tanzanian government has made progress in strengthening its health system and promoting universal health coverage, access to NCD prevention, diagnosis, and treatment remains uneven, especially among underserved and socioeconomically disadvantaged populations [12]. Structural inequities such as limited health insurance coverage, out-of-pocket payments, and geographic barriers further constrain access to care. These disparities are especially concerning given the chronic nature of NCDs and the importance of early diagnosis and long-term management. Socioeconomic status (SES) - which is a combination of economic and sociological factors, including income, education, occupation, and asset ownership - is a key social determinant of health [13]. Lower SES has been consistently linked to higher exposure to risk factors, limited access to health care, and poorer health outcomes related to NCDs [14]. For this study, asset ownership was used as the primary proxy for SES, measured through a composite wealth index constructed using principal component analysis of household assets such as housing quality, access to utilities, and ownership of durable goods. These factors shape living conditions, access to health services, and exposure to health risks. Although several studies in Tanzania have examined behavioral risk factors such as tobacco use, harmful alcohol consumption, poor diet, and physical inactivity, the role of SES in shaping NCD risk remains underexplored. A review on epidemiologic transition and the double burden of disease found that most NCD research has focused on individual-level risk behaviors, while broader social determinants, including wealth, education, and occupation, are insufficiently examined [9]. Similarly, a second work noted that many Tanzanian studies rely on facility-based samples, which limit generalizability to the broader population [15]. Therefore, this study aims to examine the relationship between household socioeconomic status and the prevalence of non-communicable diseases in mainland Tanzania. By conducting a secondary analysis of nationally representative survey data, the study contributes to the growing body of evidence on the social determinants of health in low-resource settings.

**Conceptual framework:** the conceptual framework guiding this study illustrates the relationship between independent, dependent and modifiable variables. SES measured using asset ownership is the primary independent variable. Demographic factors (age, gender, education level, marital status, residence, household size) are considered potential confounders or mediators. Health insurance serves as a modifiable variable, influencing access to services. Although not part of the statistical analysis, body mass index (BMI) is also included as a key biological intermediary pathway. Finally, the dependent variable is NCD prevalence (presence or absence of at least one NCD). This framework demonstrates how structural determinants interact with individual and health system factors to influence health outcomes.

**Objective of the study:** this study aims to determine the association between socioeconomic characteristics and NCDs, providing insights into how socioeconomic status influences NCD prevalence and health outcomes. By addressing this gap, the study seeks to inform policies and interventions that promote equity in health and healthcare delivery, ensuring that resources are allocated efficiently to support vulnerable populations. Research questions: i) what is the association between household socioeconomic status and the prevalence of NCDs in mainland Tanzania? ii) how do demographic factors mediate or confound the relationship between SES and NCD prevalence? iii) does health insurance ownership influence the likelihood of reporting NCDs across SES groups?

## Methods

**Study design and data source:** this study used secondary data from the 2021 Tanzania Willingness and Ability to Pay for Health Insurance Survey, conducted by the National Institute for Medical Research (NIMR) in collaboration with the Ministry of Health as part of efforts to inform Universal Health Coverage (UHC) policy development. The survey was a nationally representative, cross-sectional household study implemented using a stratified multistage cluster sampling design. Tanzania´s mainland was stratified into eight administrative zones, from which one representative region per zone was selected. In each region, three districts were randomly chosen, followed by purposive selection of one urban-proximal and one rural-remote ward per district. Two villages were randomly selected from each ward, and within each village, households were randomly chosen from an updated village register. This sampling frame ensured proportional representation of both rural and urban settings, geographic diversity, and socioeconomic strata. The survey was designed primarily to assess willingness and ability to pay for medical insurance, but it also collected detailed demographic, socioeconomic, and self-reported health status information, enabling secondary analyses such as the current study. The dataset is considered nationally representative because the sampling covered all mainland zones, included both rural and urban households, and applied probability sampling at each stage.

**Study setting:** data were collected from 10 regions across mainland Tanzania, representing all eight administrative zones. In each region, three districts were selected through multistage stratified random sampling, followed by purposive and simple random sampling of wards, villages, and households. The inclusion criteria for participants comprised being aged 18 years or older, residing in the selected area, and being the head of the household. While only the head of household was provided with the questionnaire, the latter was designed to capture information about all household members, ensuring that the data reflected the broader household context. Although data collection was extensive and geographically diverse, detailed documentation on the overall response rate was not publicly available, limiting full assessment of representativeness.

**Study participants:** a total of 3,566 households were included in this analysis. The unit of analysis was the household, represented by the head of household aged 18 years and above. Socioeconomic data such as asset ownership were collected at the household level, and NCD status was reported by the household head. Households were included if they had complete data on NCD status and key socioeconomic indicators. No formal sample size calculation was performed due to the secondary nature of the dataset; however, the power of the study was calculated to ensure the sample size is sufficient.

### Study variables

**Dependent variable:** presence of at least one self-reported NCD, including cardiovascular disease, diabetes, cancer, epilepsy, chronic lung disease, stomach ulcers, or hypertension. Although these conditions differ in etiology, they were grouped to reflect overall NCD burden.

**Independent variable:** Socioeconomic status, measured through an asset-based wealth index created via Principal Component Analysis (PCA), including household assets like type of roof, floor, electricity access, and ownership of items like radios and bicycles. For the purpose of this study, SES was measured using a wealth index derived from principal component analysis of household assets. The index was categorized into five quintiles: poorest, poor, average, rich, and richest. The term ‘lower SES’ refers specifically to individuals in the poorest and poor wealth quintiles.

**Confounders:** demographic variables such as age, sex, marital status, and household size were treated as confounders, based on evidence from previous studies showing their association with NCD risk. For instance, age, gender, insurance status, and household size are all known to independently affect both socioeconomic conditions and health outcomes [14,16]. Despite adjusting for known demographic and socioeconomic confounders, residual confounding remains a possibility due to unmeasured lifestyle and behavioral factors. Key variables such as tobacco use, alcohol consumption, dietary patterns, and physical activity were not captured in the dataset, potentially limiting the model´s explanatory power and ability to fully assess risk factor pathway.

**Data collection and quality:** the original data collection followed standardized, pre-tested survey instruments administered by trained enumerators. While those quality control measures enhance validity, their mention here is limited since this is a secondary analysis. This analysis makes secondary use of a dataset that was originally collected to assess willingness and ability to pay for health insurance toward achieving universal health coverage. As such, the dataset was not specifically designed for in-depth analysis of NCDs. Disease information was self-reported by household heads and lacked clinical verification or standardized diagnostic criteria, introducing potential for reporting bias and misclassification. Additionally, because the survey was not structured around disease etiology or health behavior, important NCD-related variables such as smoking, diet, and physical activity were not captured. The household response rate for the original survey was not reported; however, stratified random sampling was used to ensure representativeness across regions and population groups.

**Data analysis:** descriptive statistics were used to summarize participant characteristics. Bivariate associations between categorical variables were assessed using Pearson´s Chi-square test. Logistic regression models evaluated the relationship between SES and NCDs while adjusting for confounders. Variable selection in the multivariable model used backward elimination (p-value < 0.2), chosen to reduce model complexity by retaining only variables with stronger empirical associations while minimizing overfitting given the exploratory nature of this analysis. Backward elimination allowed a more data-driven selection suitable for secondary analysis, allowing for identification of the most impactful features without prior assumptions about which variables are important. Multicollinearity among independent variables was assessed using Variance Inflation Factor (VIF) analysis after fitting the multivariable logistic regression model. All included variables had VIF values below 10, indicating no significant multicollinearity. A post-hoc power analysis was conducted using a two-proportion comparison approach to assess whether the available sample size was sufficient to detect meaningful differences in NCD prevalence between socioeconomic groups.

**Bias:** disease information was self-reported by household heads without clinical verification or standardized diagnostic criteria. This introduces a risk of reporting bias and outcome misclassification, particularly if respondents lacked awareness of clinical diagnoses or underreported conditions due to stigma or limited health literacy. Such misclassification could bias the estimated associations between SES and NCD prevalence. Household age structure was a potential source of bias. Older individuals are more likely to report NCDs, therefore, age was included as a covariate in multivariable analysis. Access to healthcare is another source of bias. This was addressed by including health insurance ownership and residence (urban/rural) as proxy indicators in the analysis.

**Study size:** the primary dataset was collected by NIMR in 2021 using stratified multistage sampling, ensuring representativeness across Tanzania´s zones. A total of 3,566 individuals were selected based on complete case data for NCDs and SES variables. No sample size calculation was required, as all eligible records from the original survey were included in this secondary analysis.

**Ethical clearance:** ethical approval for this secondary analysis was granted by the Institutional Review Board of Muhimbili University of Health and Allied Sciences (MUHAS), approval number DA.282/298/01.C/1277, and from the National Institute for Medical Research (NIMR). Ethical approval for the original study was obtained from both the Ministry of Health and the National Institute for Medical Research.

## Results

**Participants' characteristics:** of the 3,566 households, 53.73% of the respondents were male, and 46.27% were female (Table 1). Most respondents were married and engaged in informal occupations. More than half had completed only primary education. The mean age of respondents was 38.04 years, with 45.3% aged between 18 and 34 years and 9.6% aged 55 years and above. Regarding education, 55% had completed primary education while only 15.9% had attended college or university (Table 1). Slightly more respondents resided in rural areas (53.2%) compared to urban areas (46.8%) (Table 1). A post-hoc power analysis was conducted using a two-proportion comparison approach. Assuming a baseline NCD prevalence of 13% and an expected difference of 6% between groups (equivalent to an odds ratio of approximately 1.5), a sample size of 1,170 (585 per group) would be required to achieve 80% power at a 5% significance level. Given that the study included 3,566 households, the sample size was adequate to detect meaningful differences in NCD prevalence.

**Table 1:** demographic characteristics of respondents (N = 3,566) from 10 regions across mainland Tanzania, collected in 2021 during a national cross-sectional survey by National Institute for Medical Research

Variable	Frequency (n)	Percentage (%)
**Sex**		
Male	1,916	53.73
Female	1,650	46.27
**Age group**		
18-34	1,614	45.26
35-44	986	27.65
45-54	623	17.47
55+	343	09.62
**Education**		
None	366	10.26
Primary	1,960	54.96
Secondary	673	18.87
College/University	567	15.90
**Marital status**		
Not married	1,256	36.33
Married	2,324	67.23
**Occupation**		
Formal	535	15.47
Informal	2,922	84.52
**Health Insurance status**		
Insured	606	17.53
Not insured	2,851	82.47
**Residence**		
Rural	1,897	53.20
Urban	1,669	46.80

**Prevalence of NCDs:** the prevalence of NCDs was 12.9%, with hypertension and diabetes being the most common. Only 17.53% of respondents reported having health insurance (Table 2). Prevalence was highest in the poorest quintile (24.66%) and lowest among middle-income households (10.7%). A general trend of decreasing NCD prevalence with increasing SES was observed, though not all comparisons reached statistical significance (Table 3).

**Table 2:** summary of key variables extracted for this secondary analysis from the 2021 National Household Survey conducted by the NIMR, covering 10 regions in mainland Tanzania

Variable category	Variable name	Description
**Demographic values**	Age	The age of the individual, a continuous variable that is considered a risk factor for many NCDs
	Gender	The gender of the individual (Male/Female). Gender disparities in disease prevalence have been documented
	Occupation	The type of employment or occupation held by the individual, categorized based on the nature of work (ie. formal and informal occupations).
	Education	The highest level of education completed by the individual (ie. no formal education, primary, secondary, higher education).
	Marital status	

**Table 3:** distribution of non-communicable disease (NCD) prevalence across household wealth quintiles among 3,457 households in mainland Tanzania, 2021

Wealth quintile	NCD		Total	P-value
	No (%)	Yes (%)		
Lower socioeconomic status	594 (19.73)	110 (24.66)	704 (20.36)	0.097
Low	628 (20.86)	81 (18.16)	709 (20.51)	
Average	596 (19.79)	75 (16.82)	671 (19.41)	
High	663 (22.02)	100 (22.42)	763 (22.07)	
Higher	530 (17.60)	80 (17.94)	610 (17.65)	
**Total**	3011 (100.00)	446 (100.00)	3457 (100.00)	

**Socioeconomic distribution:** based on the asset index generated through PCA, the sample was relatively evenly distributed across socioeconomic quintiles. The poorest quintile accounted for 20.36% (n = 704) of respondents, followed by the poor (20.51%, n = 709), average (19.41%, n = 671), rich (22.07%, n = 763), and richest (17.65%, n = 610). This distribution demonstrates a balanced representation of wealth categories in the study population.

**Univariate associations:** univariate analysis showed that individuals from lower SES groups had higher odds of reporting NCDs. However, after adjusting for confounders, only age, family size, and health insurance ownership remained significant. SES lost statistical significance in the final model, showing potential demographic mediators.

**Multivariable analysis:** age was found to be a strong and significant predictor of NCD prevalence, with older age groups having markedly higher odds of NCDs (Table 4). Larger family size (households with six or more members) was also associated with increased NCD prevalence (Table 4). Health insurance ownership was shown to have a protective effect against NCDs. On the other hand, place of residence and education level were not found to be significant independent predictors in the final model (Table 4). After adjusting for confounders, age, family size, and health insurance ownership remained significant. SES lost statistical significance in the final model, showing demographic mediators.

**Table 4:** crude and adjusted odds ratios from logistic regression analysis assessing the relationship between socioeconomic status and NCD prevalence, adjusting for demographic confounders

Demographic variables	Crude odds ratio			Adjusted odds ratio		
	OR	95% CI	P-value	AOR	95% CI	P-value
**SES (Poorest)**	1		1	1		
Poor	0.70	(0.51 -0.95)	0.02	0.73	(0.48 - 1.11)	0.14
Average	0.68	(0.50 - 0.93)	0.01	0.79	(0.56 - 1.11)	0.17
Rich	0.81	(0.61 - 1.09)	0.16	0.74	(0.52 - 1.05)	0.09
Richest	0.82	(0.60 - 1.11)	0.20	0.77	(0.55 - 1.09)	0.15
**Sex (male)**	1			1		
Female	1.05	(0.86 -1.28)	0.65	1.17	(0.97 - 1.41)	0.09
**Age group (<35)**	1			1		
35-44	2.13	(1.62 - 2.79)	0.00	1.95	(1.49 - 2.54)	0.00
45-54	3.01	(2.27 - 4.00)	0.00	2.69	(1.89 - 3.84)	0.00
55+	5.59	(4.11 -7.59)	0.00	5.07	(3.71 - 6.93)	0.00
**Residence (Rural)**	1			1		
Urban	1.09	(0.89 - 1.33)	0.41	1.15	(0.86 - 1.54)	0.34
**Education (None)**	1			1		
Primary	0.77	(0.56 -1.05)	0.10	1.03	(0.73 - 1.47)	0.86
Secondary	0.61	(0.42 - .90)	0.01	1.08	(0.70 - 1.69)	0.70
College/ University	0.99	(0.69 - 1.44)	0.99	1.37	(0.77 - 2.44)	0.28
**Occupation (Formal)**	1			1		
Informal	0.81	(0.63 - 1.06)	0.12	1.19	(0.72 - 1.98)	0.50
**Family size (<6 members)**	1			1		
6+ members	2.27	(1.85 - 2.79)	0.00	1.80	(1.34 - 2.42)	0.00
**Marital status (Not married)**	1			1		
Married	1.09	(.89 - 1.35)	0.40	0.89	(.70 -1.14)	0.38
**Insurance (Insured)**	1			1		
Not insured	0.59	(1.42 - 2.25)	0.00	0.68	(0.46 - 0.99)	0.05

SES: Socioeconomic status; NCD: non-communicable diseases

**Stratified analysis:** the stratified analysis further illustrated that while SES was initially associated with NCD prevalence, this relationship diminished when demographic factors were controlled for. The data show complex interactions between socioeconomic, demographic, and health system factors influencing NCD risk.

## Discussion

This study examined the relationship between SES and the prevalence of NCDs while adjusting for demographic and household-level variables using multivariable logistic regression. While the crude analysis showed a higher burden of NCDs among individuals in lower SES groups, this association was no longer statistically significant in the adjusted model. These findings suggest that the SES-NCD relationship is mediated by demographic factors such as age, family size, and insurance coverage. The results emphasize the complex interplay between structural and demographic factors in shaping NCD risk in Tanzania. The most consistent finding was the association between age and NCD prevalence. Households with older individuals were significantly more likely to report at least one NCD, which aligns with evidence from previous studies that highlight cumulative risk exposure, biological aging, and delayed access to healthcare as key contributors to chronic disease burden in low-resource settings [1,16]. In Tanzania, health systems in rural and semi-urban areas often lack preventive services tailored for older populations [10]. This underscores the importance of expanding screening programs and geriatric services to mitigate age-related health risks. Family size also emerged as a significant predictor of NCD burden. Households with six or more members had higher odds of reporting an NCD. This may be linked to economic constraints in large families, which can limit the ability to afford nutritious food, routine checkups, or medication. The Food and Agriculture Organization (FAO) has documented how limited access to diverse micronutrients in lower SES, high-dependency households contributes to malnutrition and metabolic disorders [17]. Targeted nutritional interventions and social protection measures for large families could help reduce this risk and improve overall health resilience.

The study also found that health insurance coverage was significantly associated with lower odds of reporting NCDs. This protective effect likely reflects improved access to preventive and curative health services among the insured. However, it is also possible that individuals only enroll in insurance schemes after experiencing health problems, indicating reactive rather than proactive enrollment patterns. This observation is supported by findings from Kagaigai *et al*. (2023), who observed that individuals with chronic illnesses are more likely to seek insurance after diagnosis. This reactive pattern of insurance uptake indicates missed opportunities for early prevention. Promoting broader and earlier enrollment in national insurance schemes such as the Community Health Fund (CHF) or improved CHF (iCHF) could help ensure timely access to diagnostic services and continuity of care [18]. Insurance reforms should also consider integrating NCD screening into basic benefit packages and subsidizing premiums for high-risk households. Strengthening insurance outreach programs and creating incentives for early enrollment, particularly among younger, healthier individuals can improve health outcomes and reduce future treatment costs.

The descriptive results revealed a surprising pattern, that is, a similar NCD prevalence among the lower and highest SES quantiles. This non-linear distribution suggests that both under diagnosis in low-resource households due to limited health access and lifestyle-driven NCD risks in wealthier households (e.g., sedentary behavior, processed food consumption, stress) may coexist. This result deserves further exploration using disease-specific analyses and qualitative data. It challenges the conventional assumption of a linear socioeconomic gradient in health outcomes and points to the complexity of NCD epidemiology in transitional economies like Tanzania. Taken together, these findings highlight the importance of interpreting SES-NCD associations in context. While global evidence supports the role of socioeconomic inequalities in shaping chronic disease risk, the influence of age, household structure, and insurance coverage in this Tanzanian sample suggests that broader demographic transitions and health system access play a more immediate role in determining NCD outcomes. This underscores the need for multi-layered policies that go beyond income or asset-based targeting. Policy recommendations should focus on expanding insurance enrollment, integrating NCD screening into primary care services, and increasing outreach to large, aging, and underserved households. However, these interventions must be realistic given Tanzania´s resource constraints. Task-shifting to community health workers, mobile health technology, and integration of NCD care into maternal and child health platforms may offer cost-effective solutions. Importantly, all strategies must be aligned with Tanzania´s National Strategic Plan for NCDs and existing health financing reforms.

**Limitations and future research:** several limitations should be noted. The study relied on self-reported NCD data from household heads, which may introduce recall or misclassification bias. Additionally, the dataset lacked variables on behavioral risk factors such as smoking, alcohol use, diet, and physical activity limiting a more comprehensive understanding of causal pathways. Finally, the cross-sectional design precludes causal inference and limits our ability to assess temporal relationships between SES and NCD outcomes

**Interpretation:** the study supports the hypothesis that socioeconomic inequality significantly contributes to the NCD burden. Demographic and household-level factors interact with SES in determining NCD risk.

**Generalizability:** the dataset used stratified multistage random sampling, ensuring coverage across all administrative zones and rural/urban strata. By including 10 regions and more than 3,500 households, the sampling frame captured broad geographic, economic, and demographic diversity. This structured and proportional sampling enhances the external validity and supports the generalizability of findings to the adult household population of mainland Tanzania.

## Conclusion

This study reveals lower SES is associated with increased prevalence of NCDs. Demographic factors such as age, family size, and gender also play a critical role in the NCD risk, and the complex interactions between SES, demographic characteristics, and NCD prevalence warrant tailored public health interventions. Public health interventions should be socioeconomically targeted and take into account household demographics and access barriers. Finally, future research should consider longitudinal methods.

### What is known about this topic

- Socioeconomic status influences the burden and management of non-communicable diseases globally;
- Lower socioeconomic groups face higher barriers in accessing preventive and curative services for non-communicable;
- Disparities in awareness and affordability impact non-communicable diseases diagnosis and control.

### What this study adds

- Non-communicable diseases prevalence is higher among lower socioeconomic status households in Tanzania, as shown using national representative data;
- Demographic and health system variables influence the socioeconomic status -non-communicable diseases association;
- Health insurance ownership correlates with non-communicable diseases presence, indicating potential delayed enrollment post-diagnosis.
